# American Dental Association

**Published:** 1897-10

**Authors:** 


### Reports of Society Meetings.

### AMERICAN DENTAL ASSOCIATION.

(Continued from page 588.)

*August 3, 1897.—First day.—Evening Session.*

THE meeting was called to order by the vice-president, Dr. Fillebrown.

The secretary read the minutes of the morning session, which were approved.

Dr. C. N. Peirce moved that the President's address be referred to a committee of five for consideration. Motion carried.

The chairman appointed Drs. J. D. Patterson, H. B. Noble, F. G. Dunbar, H. G. Burkhart, and Grant Molyneaux.

The secretary moved that the treasurer be instructed to pay all properly authenticated bills. Motion carried.

Section IV., Histology and Microscopy.

Owing to a delay in the report this section was passed for the time being.

Section V., Materia Medica and Therapeutics.

Dr. Cassidy reported that the section had a paper by Dr. Weston A. Price, of Cleveland, Ohio, on "The Physiology of Cataphoresis."

Dr. Emma Eames Chase, corresponding secretary, reported as follows:

At the request of the president, notices of this meeting were sent to each dental society and dental journal, calling attention to the importance of the meeting, and reminding each society that it is entitled to one representative for every five members, and asking that delegates properly instructed be sent to this meeting. Blank certificates have been mailed to all societies requesting them.

### COMMITTEE ON UNION.

*Dr. Fillebrown.*—I wish to say that we have considered this subject, and have met with the other committee. The opinion of this committee is unanimous; they are prepared to present the report at the proper time, and I move that to-morrow evening the consideration of the report of this Committee on Union be made the first order of business. Motion carried.

Dr. Price then read a paper entitled, "The Physiology of Cataphoresis."

(For Dr. Price's paper, see page 647.)

DISCUSSION.

*Dr. Rhein.*—It has been a great pleasure for me to listen to the very able paper we have had on this subject. As far as my investigations with the cataphoric diffusion of medicinal agents have gone, they bear out entirely what the author of this paper has presented to us this evening. The only point of issue that I could perceive in what he has given us is the question of the means by which the medicinal agents are passed through the different substances,—whether it is by direct physical pressure or by the liberation of some of the molecular elements. I do not intend to discuss that portion of the paper, because it is necessary to follow it up by further investigations before the truth or the incorrectness of this theory can be established. I have been making some investigations along that line, although in a different channel, in order to produce, if possible, anæsthesia of the dentine and pulp by osmotic pressure, without the electric current; but I have not proceeded far enough to form any conclusions as yet. There is no question but what, if the pressure theory is a correct one, we will be able to obtain the anæsthetic effects by other means than by the electrical powers alone. It is the practical results that this paper has given us that interested me principally. I refer to the stress the author laid on the use of a milliampèremeter in cataphoresis. From investigations in different sections of the country as to the manner in which the different instruments were used, and the way in which they were endeavoring to sell them to the profession, I have learned that many men are trying to use this very valuable means without the use of a milliampèremeter. That cannot be too strongly condemned, for the excellent reason the author has given us. The other interesting point from a practical view is the ratio of data which he gives us from the numerous cases that he has so carefully observed, and the reading of those cases *seriatim* will certainly be of great interest to us. My own impression is that the solution of a reduction of the time limit resolves itself to a great extent in our ability to increase the amount of potential force that we introduce, and at the same time increase the amount of ampèrage that is forced through the tooth. I think the means of accomplishing that will be found in an improvement in the anode ends that are introduced into the cavity itself. I think the great mistake has been the use of points too narrow for the diameter that was expected to be anæsthetized, which renders it ineffectual to diffuse a sufficient amount of current in a given space, and that the end of the anode should be as large as it is possible to introduce in a given-sized cavity. This is the line which, if followed out properly, I believe, will enable us to reduce the time limit. We have all heard of the anæsthetization of dentine in a very few minutes, and whenever I have endeavored to follow up any such experimentation by personal investigation I have found that the amount of surface that was anæsthetized was not sufficient to produce complete anæsthesia, due very possibly to the imperfect cleansing of carious cavities that some of the profession is accustomed to.

*Dr. Custer.*—The paper is one which every one of us should look up when it appears in print. It is worthy of study and consideration, and is a monumental paper upon the question now before the profession as to the physiological action of cataphoresis.

*Dr. Cassidy.*—I endorse the stand that Dr. Price has taken with respect to the action of drugs cataphorically depending upon electrolysis. The point that struck me as rather novel in the paper, as proved by experiment, was that sensory nerves were poorer conductors than the muscular tissue, and that the nerve portion of a pulp is less affected by the current than the other constituents. In regard to the resistance between the two poles of the current, when there is a current passed between them those two points are poles no longer, and we take it for granted, it seems, thus far, that it is the anode or positive pole that must be presented in connection with the medicine.

If we wish to use cataphoresis in bleaching teeth, say with peroxide of hydrogen, and we want the full effect of the cataphoric action, instead of using the positive pole better results will be obtained by using the negative, because the hydrogen will seek the opposite pole. It will penetrate more and oxidize the substance causing the discoloration of the tooth more rapidly and more effectively than if the positive pole is used. The oxygen will not react any better than without the current until it receives a higher potential than the positive pole itself. The electrolytes, as the paper stated, must be compound in their nature, and must be in a fluid condition, in order to act. A mere solution of iodine is not a good electrolyte, but a compound of iodine with some element like potassium makes an excellent electrolyte. In such a case the laws of electrolysis are called into play. The iodine will go towards the positive pole, therefore the negative pole should be the one used in connection with potassium iodide. You get better results from it. An element such as chlorine is not an electrolyte at all. It would be the water (if it be an aqueous solution) that would act. In using sulphuric acid to penetrate farther than by mere application, it is not the ion that affects the tissues. The sulphuric acid is decomposed, the sulph-ion ($SO_4$) penetrates, and it meets at the same time with the positive ion of water,—namely, hydrogen. What is the consequence? Decomposition of the acid by the electric current sets free hydrogen and the sulph-ion, $SO_4$. The hydrogen in a solution of that kind will escape at the negative pole, the sulph-ion at the positive, and when so escaping the sulph-ion takes up what hydrogen there is in the water and forms sulphuric acid, and acts on the tissues.

*Dr. St. George Elliot.*—I endorse all that has been said in regard to the very great value of this paper, for the reason that the gentleman who wrote it has taken the trouble to go very deeply into the matter. Most of us are quite satisfied to make practical demonstrations of certain theories, which we formulate in our own minds, and then we make certain practical applications of those theories; but there are very few of us competent to go into the matter as has the author of this paper; to properly discuss it we ought to read and study it and experimentally examine the facts.

*Dr. Price.*—I would like to say a word about the question that was raised, whether the nature of the electrodes would aid at all in shortening the time. The time is dependent upon the pain limit. It will not make a particle of difference what combinations we get in the circuit of a constant current; the same amount of current flowing through that nerve will produce the same result. I have had a man shut his eyes, and when I applied the current, he would tell me when a certain amount was reached every time, within the two hundred-thousandth part.

Adjournment.

(To be continued.)

